# Editorial: Special Issue on “The Role of Exosomes in Cancer Diagnosis and Therapy”

**DOI:** 10.3390/ijms241813716

**Published:** 2023-09-06

**Authors:** Nils Ludwig, Torsten E. Reichert

**Affiliations:** Department of Oral and Maxillofacial Surgery, University Hospital Regensburg, 93053 Regensburg, Germany

As a result of extensive research in recent years, small extracellular vesicles (sEVs), also known as exosomes, are now considered major contributors to intercellular communication in health and disease. sEVs carry a complex cargo composition consisting of proteins, nucleic acids, and lipids, and it has been shown that components of sEVs are biologically active and induce effects in recipient cells. These characteristics of sEVs motivated researchers worldwide to further explore their biology under physiological as well as pathophysiological conditions. Tumor cell-derived sEVs in particular have been extensively studied. It was demonstrated that tumor cell-derived sEVs promote tumor progression through local and systemic effects and that they functionally contribute to malignant processes such as metastasis, immunosuppression, angiogenesis, and drug resistance. Besides their functional effects in the tumor microenvironment (TME), sEVs have been shown to serve as attractive biomarkers and to have potential for utilization in sEV-based nanotherapeutic approaches. These versatile roles of sEVs in cancer are highlighted in this Special Issue, which is a collection of articles fully dedicated to sEV biology.

One focus of this Special Issue is the role of sEVs as clinical biomarkers and the article by Paluschinski et al. presents an approach to identify sEV-based biomarkers in liver diseases [1]. The optimized workflow contains all the steps of sEV analysis including isolation, characterization, cargo analysis, and, ultimately, biomarker validation. A comparison of different groups of patients with nonalcoholic fatty liver disease (NAFLD), autoimmune hepatitis (AIH), intrahepatic cholangiocarcinoma (CCA), and respective healthy volunteers revealed that microRNAs (miR-10a, miR-21, miR-142-3p, miR-150, and miR-223) as well as cytokines (IL2, IL8, and IFNγ) enriched in sEVs can be utilized to discriminate between these liver diseases. Thus, the authors present an optimized approach to systematically screen for the sEV-based biomarkers related to disease diagnosis and prognosis, which has the potential to accelerate the implementation of personalized treatment strategies. The concept of using sEVs as biomarkers in liver diseases, and particularly as predictive biomarkers for treatment response and survival, was also supported by the article by Gylstorff et al., which characterizes the immunological profile of sEVs in the blood of patients with inoperable hepatocellular carcinoma (HCC) [2]. Here, the profiles of sEVs were assessed before and after selective internal radiotherapy (SIRT). This comprehensive article first describes the characteristics of the 50 patients included in the study and presents the clinical characteristics related to the treatment and outcomes. Alterations in blood counts, cytokine levels, and immunological markers in plasma-derived sEVs were analyzed in blood samples collected prior to and after SIRT and several markers were identified which correlated with patient response and survival. Here, plasma-derived sEVs carried a broad range of markers related to the immune system, cancer, or coagulation. Some markers, such as CD4, positively correlated with therapy response and survival, whereas other markers, such as CD20, CD49e, and CD146, were negatively associated with survival. Gylstorff et al. [2] demonstrated that the sEV landscape and cargo composition in the blood are promising predictive biomarkers of response and can be utilized for monitoring HCC patients undergoing SIRT.

Similar to the study by Paluschinski et al. [1], the article published by Van Hoof et al. [3] presents an optimized workflow for isolating and analyzing sEVs for cancer diagnostics. Specifically, this study was dedicated to exploring the intravesicular DNA content of sEVs and, therefore, addresses a topic which is controversial in the literature. While it was shown that DNA templates can be attached to the surface of sEVs, whether DNA can also be found in the lumen of sEVs is still under investigation. Van Hoof et al. [3] demonstrated that sEVs mainly enclose short genomic DNA (<35–100 bp) and that this DNA can be used to detect the *EGFR* T790M mutation in sEVs isolated from non-small-cell lung cancer cells. The authors detected significantly higher mutant allele frequencies in comparison to standard cell-free DNA extraction, which was, in part, due to the co-purification of circulating tumor DNA. This is a significant finding since looking at intravesicular DNA might be another promising clinical cell-free DNA process which might be used alone or in tandem with an analysis of the circulating tumor DNA, ultimately improving and simplifying cancer genotyping.

The study by Patel et al. [4] focusses on oral squamous cell carcinoma (OSCC) and, while most of the published literature on OSCC-derived sEVs analyzes sEVs isolated from the blood of OSCC patients, the source of sEVs in the study of Patel et al. was saliva. By comparing sEVs isolated from the tumor tissues to sEVs isolated from the saliva of OSCC patients or healthy donors, the authors demonstrated that miR-1307-5p was exclusively overexpressed in the tissue and salivary sEVs of OSCC patients compared to the sEVs isolated from healthy donors. Therefore, sEV-associated miR-1307-5p was introduced as a novel diagnostic tool in OSCC.

Matthiesen et al. contributed an article to this Special Issue looking at sEVs in diffuse large B cell lymphoma (DLBCL) [5]. Mass spectrometry was used for the sEV analysis and the authors identified protein signatures which are uniquely present in sEVs isolated from 32 DLBCL patients, comparing these to sEVs isolated from 15 age-matched healthy donors. These protein signatures could thus effectively identify DLBCL patients. Also, Matthiesen et al. were able to detect protein signatures which significantly correlated with patient survival. The study gives major insights into the proteome of sEVs in DLBCL and contributes to the establishment of novel cancer biomarkers by providing comprehensive mass spectrometry data and also by presenting a process which could be used in large-scale patient cohorts.

One article of this Special Issue is dedicated to the therapeutic aspects of sEVs. Milutinovic et al. investigated the role of sEVs isolated from human-bone-marrow-derived mesenchymal stromal cells (MSCs) in restoring cisplatin-induced cognitive impairments and brain damage [6]. The authors demonstrated in vivo that the intranasal delivery of MSC-derived sEVs reversed cisplatin-induced deficits in executive function and working as well as spatial memory. A thorough analysis of the mouse model revealed that this effect was based on structural changes in the mitochondria, white matter, and hippocampus, and that it was orchestrated by several molecular pathways, including netrin and Wnt/Ca^2+^ signaling.

The Special Issue also contains two comprehensive reviews which focus on the role of sEVs in breast cancer [7] as well as malignant mesothelioma (MM) [8]. The review dedicated to breast cancer was written by Loric et al. and it summarizes the most recent literature on the role of sEVs in normal and cancerous breast tissues [7]. The focus of the review was to evaluate the use of sEVs as a novel source of liquid biopsy for cancer diagnosis, follow-up, and prognosis. The role of sEVs in breast cancer treatment, either as novel therapeutic targets or as efficient drug delivery vehicles, is also summarized. The second review, written by Munson and Shukla, focusses on sEVs in MM and summarizes the research data of the authors as well as the recently published literature [8]. So far, there is only a limited number of studies on sEVs in MM available; therefore, this review is of great value to the field and introduces the characteristics and functions of sEVs in MM and summarizes all the current sEV-related studies in a comprehensive manner. The overview is helpful for the overall understanding of sEV-related aspects in MM and has the potential to accelerate research progress in this particular field.

The aim of this Special Issue was to examine the use of sEVs for cancer diagnosis and therapeutics. The collection of articles demonstrates the versatility of sEV biology and adopts an interdisciplinary approach to further enhance our understanding of the role of sEVs in cancer. The articles cover different malignant entities, including CCA, HCC, lung cancer, OSCC, DLBCL, breast cancer, and MM, and also cover the analysis of different components of sEVs including proteins, DNA, and miRNA. The articles present several novel processes for sEV analysis which may accelerate the screening for sEV-based biomarkers and lead to even more sensitive approaches with the potential to be translated into clinical use. We hope that this collection is of great value to the sEV community and that it will inspire and facilitate the implementation of future sEV-based studies. We conclude this introduction by expressing our gratitude to the *International Journal of Molecular Sciences* and its reviewers for their support and insightful contributions to this Special Issue.

## References

[1] Paluschinski M., Loosen S., Kordes C., Keitel V., Kuebart A., Brandenburger T., Schöler D., Wammers M., Neumann U.P., Luedde T. (2023). Extracellular Vesicles as Markers of Liver Function: Optimized Workflow for Biomarker Identification in Liver Disease. Int. J. Mol. Sci..

[2] Gylstorff S., Wilke V., Kraft D., Bertrand J., Pech M., Haag F., Relja B. (2023). Selective Internal Radiotherapy Alters the Profiles of Systemic Extracellular Vesicles in Hepatocellular Carcinoma. Int. J. Mol. Sci..

[3] Van Hoof R., Deville S., Hollanders K., Berckmans P., Wagner P., Hooyberghs J., Nelissen I. (2022). Intravesicular Genomic DNA Enriched by Size Exclusion Chromatography Can Enhance Lung Cancer Oncogene Mutation Detection Sensitivity. Int. J. Mol. Sci..

[4] Patel A., Patel S., Patel P., Mandlik D., Patel K., Tanavde V. (2022). Salivary Exosomal miRNA-1307-5p Predicts Disease Aggressiveness and Poor Prognosis in Oral Squamous Cell Carcinoma Patients. Int. J. Mol. Sci..

[5] Matthiesen R., Gameiro P., Henriques A., Bodo C., Moraes M.C.S., Costa-Silva B., Cabeçadas J., da Silva M.G., Beck H.C., Carvalho A.S. (2022). Extracellular Vesicles in Diffuse Large B Cell Lymphoma: Characterization and Diagnostic Potential. Int. J. Mol. Sci..

[6] Milutinovic B., Mahalingam R., Mendt M., Arroyo L., Seua A., Dharmaraj S., Shpall E., Heijnen C.J. (2023). Intranasally Administered MSC-Derived Extracellular Vesicles Reverse Cisplatin-Induced Cognitive Impairment. Int. J. Mol. Sci..

[7] Loric S., Denis J.A., Desbene C., Sabbah M., Conti M. (2023). Extracellular Vesicles in Breast Cancer: From Biology and Function to Clinical Diagnosis and Therapeutic Management. Int. J. Mol. Sci..

[8] Munson P., Shukla A. (2022). Potential Roles of Exosomes in the Development and Detection of Malignant Mesothelioma: An Update. Int. J. Mol. Sci..

